# Programmed necrosis - a new mechanism of steroidogenic luteal cell death and elimination during luteolysis in cows

**DOI:** 10.1038/srep38211

**Published:** 2016-11-30

**Authors:** Takuo Hojo, Marta J. Siemieniuch, Karolina Lukasik, Katarzyna K. Piotrowska-Tomala, Agnieszka W. Jonczyk, Kiyoshi Okuda, Dariusz J. Skarzynski

**Affiliations:** 1 Institute of Animal Reproduction and Food Research, Polish Academy of Sciences, Olsztyn, 10-748, Poland; 2 Graduate School of Environmental and Life Science, Okayama University, Okayama, 700-8530, Japan; 3 Obihiro University of Agriculture and Veterinary Medicine, Hokkaido, 080-8555, Japan

## Abstract

Programmed necrosis (necroptosis) is an alternative form of programmed cell death that is regulated by receptor-interacting protein kinase (RIPK) 1 and 3-dependent, but is a caspase (CASP)-independent pathway. In the present study, to determine if necroptosis participates in bovine structural luteolysis, we investigated RIPK1 and RIPK3 expression throughout the estrous cycle, during prostaglandin F2α (PGF)-induced luteolysis in the bovine corpus luteum (CL), and in cultured luteal steroidogenic cells (LSCs) after treatment with selected luteolytic factors. In addition, effects of a RIPK1 inhibitor (necrostatin-1, Nec-1; 50 μM) on cell viability, progesterone secretion, apoptosis related factors and RIPKs expression, were evaluated. Expression of RIPK1 and RIPK3 increased in the CL tissue during both spontaneous and PGF-induced luteolysis (P < 0.05). In cultured LSCs, tumor necrosis factor α (TNF; 2.3 nM) in combination with interferon γ (IFNG; 2.5 nM) up-regulated *RIPK1* mRNA and protein expression (P < 0.05). TNF + IFNG also up-regulated *RIPK3* mRNA expression (P < 0.05), but not RIPK3 protein. Although Nec-1 prevented TNF + IFNG-induced cell death (P < 0.05), it did not affect *CASP3* and *CASP8* expression. Nec-1 decreased both RIPK1 and RIPK3 protein expression (P < 0.05). These findings suggest that RIPKs-dependent necroptosis is a potent mechanism responsible for bovine structural luteolysis induced by pro-inflammatory cytokines.

In many species, the corpus luteum (CL) is a transient endocrine gland responsible for the secretion of progesterone (P4), a sex steroids that is essential for establishment and maintenance of pregnancy^1^. When animals do not become pregnant, regression of the CL, called luteolysis, is essential for normal cyclicity because it allows the development of new follicles. Luteolysis in ruminant species results from the uterine release of prostaglandin F2α (PGF)^2^. In cattle, luteolysis can also be pharmacologically induced by administration of PGF analogs by injection. Luteolysis involves reduction in P4 production (functional luteolysis) and tissue degradation by cell death (structural luteolysis)^3^,^4^. Generally, caspase-dependent apoptosis, also known as type I programmed cell death^5^, in the cells that form the CL, such as luteal steroidogenic cells (LSC) and luteal endothelial cells (LEC), is thought to be the predominant pathway for cell death during luteolysis in several species including cattle^3^,^4^. A large number of factors have been implicated in structural and functional regression of bovine CL^6^,^7^,^8^,^9^,^10^,^11^. Apoptosis of luteal cells and CL vascular regression are regulated/modulated by pro-inflammatory cytokines, i.e., tumor necrosis factor-α (TNF), interferon-γ (IFNG), FAS ligand (FASL) and nitric oxide (NO)^6^,^7^,^8^,^9^,^10^. On the other hand, P4, cortisol and luteotropic PGs (PGE2 and PGI2) protect LSCs against apoptosis^11^,^12^,^13^. As mentioned above, apoptosis in CL is regulated by complex mechanisms inducing a cascade of several immune-endocrine factors and mediators.

Apoptosis occurs through two main signaling cascades, which are known as the death receptor pathway and the mitochondrial pathway^14^,^15^. The death receptor pathway, which is also known as the extrinsic apoptotic route, is initiated by extracellular signals (e.g., FasL, TNFα) that interact with cell surface receptors (e.g., Fas, TNFRs) that are responsible for transduction of cell death signaling^14^. On the other hand, the mitochondrial pathway, which is also called the intrinsic apoptotic cascade, is regulated by members of the Bcl-protein family. The relative ratio of Bcl-2, which protects against cell death, and Bax, a proapoptotic protein, determines cell fate^15^. These two pathways are not completely separated and share part of signals. In fact, both of these pathways are characterized by activation of caspases (CASPs), which are intracellular cysteine aspartic proteases^16^. Death ligands, such as TNF and FASL, when bound to the death receptor expressed on cell membranes, can activate an upstream CASP named CASP8. CASP8 (activated CASP) induces downstream executor CASPs including CASP3, finally resulting in DNA fragmentation and apoptosis^17^. During the past several decades, apoptosis is considered to be the most studied local mechanism regulating structural luteolysis of the bovine CL. Thus, a great number of studies have been focused on type-I programmed cell death of steroidogenic and accessory luteal cells^6^,^7^,^8^,^9^,^10^,^11^,^12^,^13^. However, luteolysis, especially exogenous PGF-induced luteolysis, is a very rapid process and the bovine CL completely disappears from the ovary within 2 days after PGF injection. Thus, one may consider that apoptosis alone is not a sufficient mechanism to induce this acute luteolysis.

There are also caspase-independent cell death pathways, which are involved in homeostasis of different tissues and organs^18^. Necrosis is one cell death mechanism known to be caspase-independent. Generally, necrosis has been considered as an accidental and undesirable cell demise pathway. Furthermore, it is carried out in a non-regulated manner and caused by extreme conditions. However, recently it was reported that necrosis can be regulated by intracellular mechanisms^19^. When apoptosis is blocked by caspase inhibitors such as zVAD-FMK, the necroptosis pathway, which is an alternative cell death pathway, is activated^18^,^20^,^21^. Receptor-interacting protein kinase (RIPK) 1 and RIPK3 are known to play roles as sensors of cellular stress^22^ and are essential kinases mediating the programmed necrosis pathway^21^,^23^,^24^,^25^. Moreover, death ligands such as TNF and Fas ligand induce not only apoptosis but also necrosis in a number of tissues^26^,^27^. RIPK1 binds to members of the TNF receptor super family such as TNFR1, FAS, tumor necrosis factor-related apoptosis-inducing ligand receptor (TRAILR) 1 and TRAILR2, and is crucial in the necroptotic pathway induced by these TNF receptor super family members^26^. Indeed, RIPK3 is also known to be a necessary modulator for necroptosis^23^,^24^,^25^,^27^. There are several reports about necroptosis mediated by TNF receptor (TNFR) type 1^25^,^27^,^28^, and it is also reported that TNFR1 and FAS, which are members of the TNF receptor super family, play important roles in cell death in bovine LSCs^7^. Therefore, we hypothesized that necroptosis occurs in bovine LSC and contributes to luteolysis. However, it is still unclear whether necroptosis is one of the mechanisms responsible for structural regression of the bovine CL.

In the present study, to test the hypothesis that necroptosis serves as a necessary and/or accessory mechanism for CL cell death during luteolysis in the cow, we investigated: (1) the expression of RIPK1 and RIPK3 in bovine CL tissues throughout the estrous cycle and during PGF-induced luteolysis *in vivo*, (2) the local regulatory mechanism of RIPKs expression in luteal cells, and (3) the participation of RIPKs in LSC death mechanisms by using an *in vitro* cell culture system with necrostatin-1 (Nec-1), an allosteric inhibitor of RIPK1 activity.

## Results

### Changes in RIPK1 and RIPK3 mRNA and protein expression throughout the estrous cycle and PGF-induced luteolysis *in vivo*

**Figure 1:**
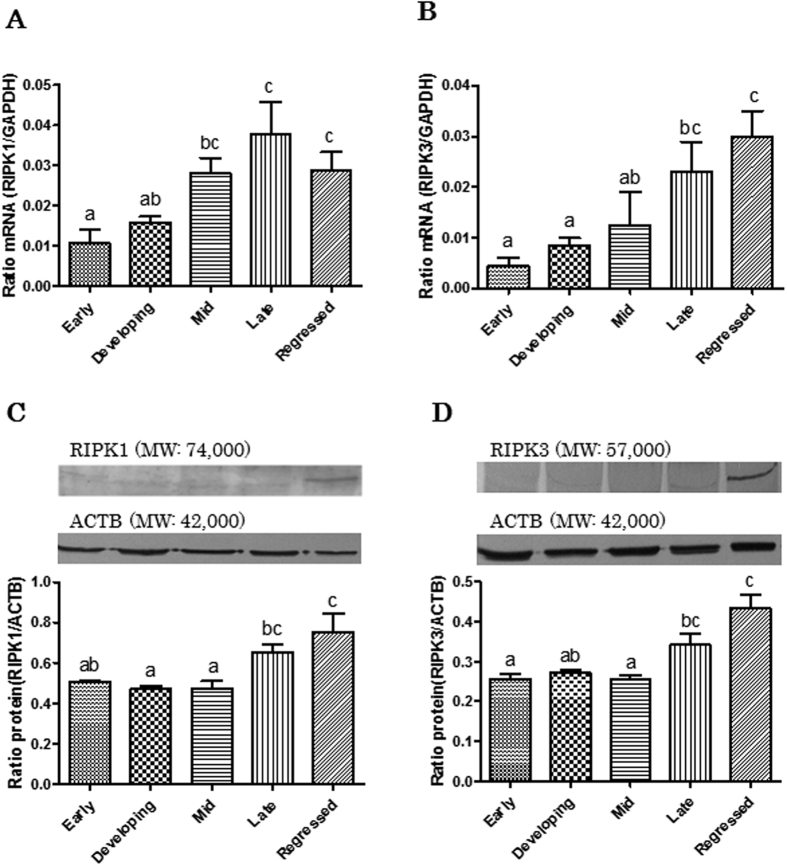
Changes in the relative amounts of RIPK1 and RIPK3 mRNA and protein expression in the bovine CL tissues throughout the estrous cycle. (**A**) and (**B**) Comparison of relative amounts of RIPK1 or RIPK3 mRNA determined by quantitative RT-PCR in bovine CL tissue throughout the estrous cycle (early: Days 2–3; developing: Days 5–6; mid: Days 8–12; late: Days 15–17; regressed luteal stages: Days 19–21). Data are the mean ± SEM for four samples/stage and are expressed as the relative ratio of RIPK1 or RIPK3 mRNA to GAPDH mRNA. (**C**) and (**D**) Representative western blot bands for RIPK1 or RIPK3 and ACTB. Densitometrically analyzed western blot results in bovine CL tissue during different luteal phases. Data are the mean ± SEM for four samples/stage and are expressed as the relative ratio of RIPK1 or RIPK3 protein to ACTB protein. Different superscript letters indicate significant differences (P < 0.05), as determined by non-parametric Kruskal-Wallis test followed by Dunnett’s multiple comparison test.

**Figure 2:**
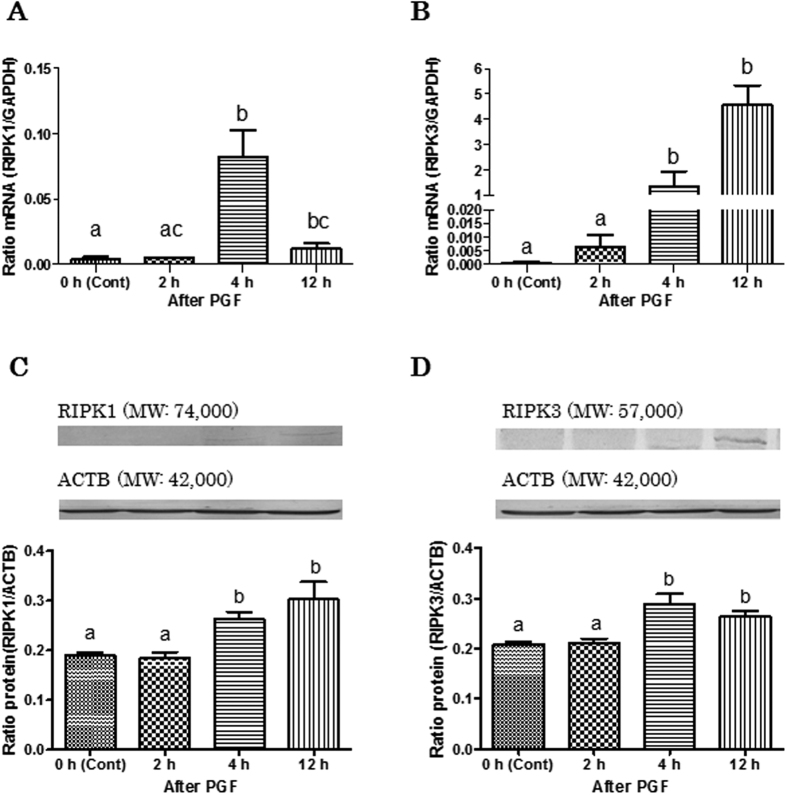
Changes in the relative amounts of RIPK1 and RIPK3 mRNA and protein expression in the bovine CL tissues during PGF-induced luteolysis. (**A**) and (**B**) Comparison of relative amounts of RIPK1 or RIPK3 mRNA determined by quantitative RT-PCR in bovine CL tissue after PGF administration (0 h: control; 2 h, 4 h and 12 h). Data are the mean ± SEM for four samples/stage and are expressed as the relative ratio of RIPK1 or RIPK3 mRNA to GAPDH mRNA. (**C**) and (**D**) Representative western blot bands for RIPK1 or RIPK3 and ACTB. Densitometrically analyzed western blot results in bovine CL tissue during different luteal phases. Data are the mean ± SEM for four samples/stage and are expressed as the relative ratio of RIPK1 or RIPK3 protein to ACTB protein. The data were statistically analyzed by a non-parametric Kruskal-Wallis test followed by Dunnett’s multiple comparison test. Different superscript letters indicate significant differences (P < 0.05).

To clarify the presence of necroptosis, the expression of necroptosis main inducer RIPK1 and RIPK3 in bovine CL tissues throughout the estrous cycle and during PGF-induced luteolysis were investigated by real-time PCR and western blotting. Real-time PCR analysis showed that expression of *RIPK1* and *RIPK3* mRNA in bovine CL tissues was present throughout the luteal stages of the cycle and was estrous cycle dependent. *RIPK1* mRNA expression (*RIPK1* mRNA/*GAPDH* mRNA ratio) increased after the mid luteal stage until regression (Fig. 1A; P < 0.05) and the highest *RIPK3* mRNA expression was found at the regressed luteal stage (Fig. 1B; P < 0.05). Expression of both RIPK1 and RIPK3 protein was low during early to mid luteal stages, and increased significantly at the late luteal stage (Fig. 1C and D; P < 0.05). *RIPK1* and *RIPK3* mRNA expression levels were higher 4 h and 12 h after PGF injection (Fig. 2A and B; P < 0.05), compared to the control. RIPK1 and RIPK3 protein expression was higher 4 h after PGF injection than in the control (Fig. 2C and D; P < 0.05). Full length lanes of western blotting are shown in Supplementary Fig. 3.

**Figure 3:**
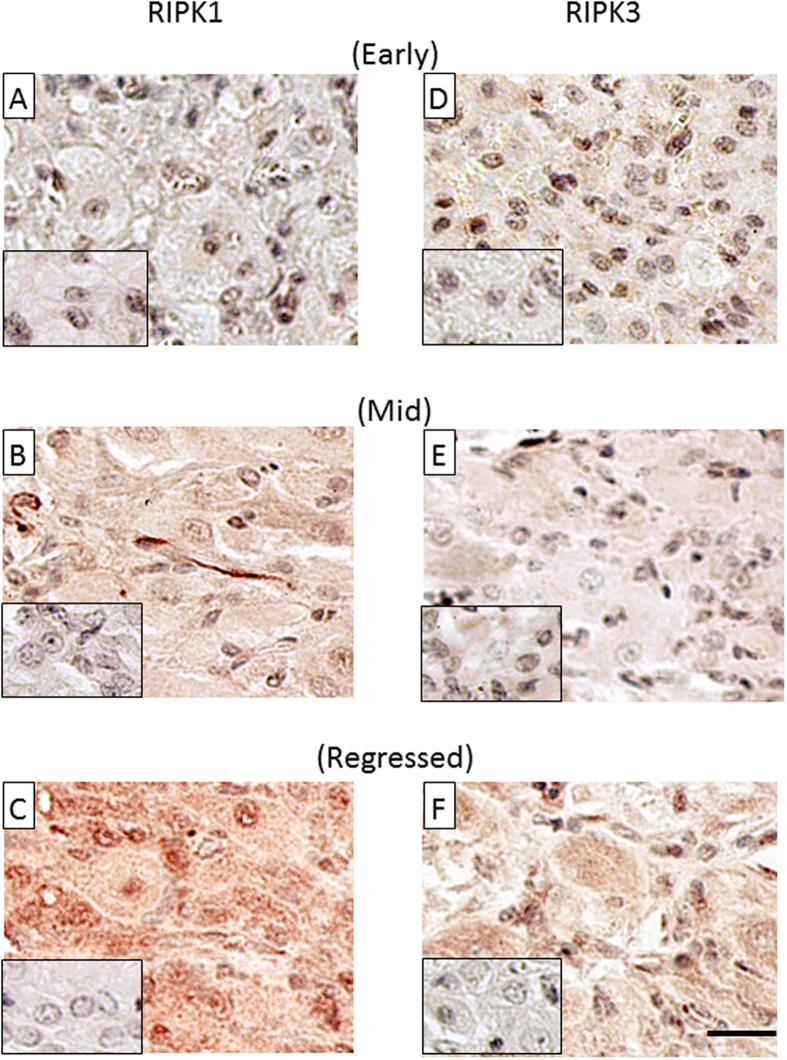
Localization of RIPK1 and RIPK3 protein in the bovine CL tissues throughout the estrous cycle. Representative images of localization of RIPK1 (**A–C**) and RIPK3 (**D–F**) protein in bovine CL tissues throughout the estrous cycle. Each small window shows a negative control stained with normal rabbit IgG instead of primary antibody. Bar = 25 μm.

**Figure 4:**
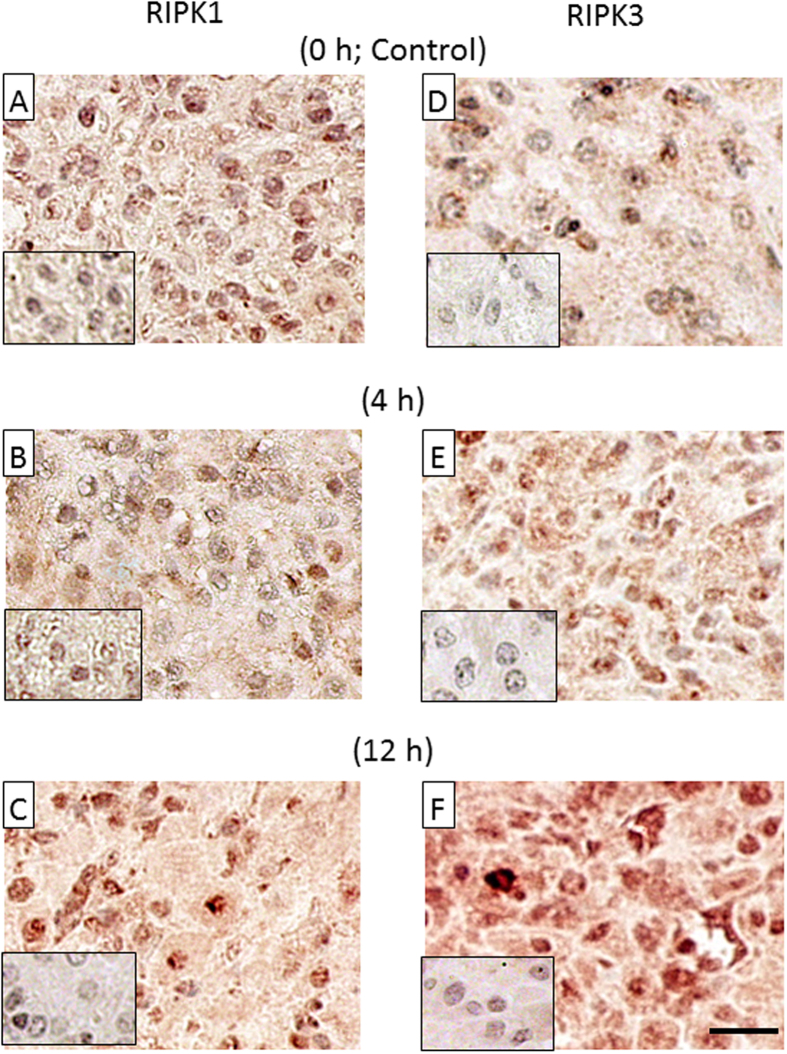
Localization of RIPK1 and RIPK3 protein in the bovine CL tissues during PGF-induced luteolysis. Representative images of localization of RIPK1 (**A–C**) and RIPK3 (**D–F**) protein in bovine CL tissues during PGF-induced luteolysis. Each small window shows a negative control stained with normal rabbit IgG instead of primary antibody. Bar = 25 μm.

Then, to investigate the localization of RIPK1 and RIPK3 in bovine CL tissue, immunohistochemistry was performed. For both RIPK1 and RIPK3 antibodies, whole tissue, especially luteal endothelial cells (arrowhead), showed positive staining at the regressed luteal stage and 12 h after PGF injection (Figs 3C,F and 4C,F).

### Expression of RIPK1 and RIPK3 in LSCs and LECs, and local regulatory mechanisms of RIPK1 and RIPK3 expression in cultured LSCs

As a preliminary study, *RIPK1* and *RIPK3* mRNA expression was confirmed by RT-PCR in isolated LSCs (Supplementary Fig. 1).

**Figure 5:**
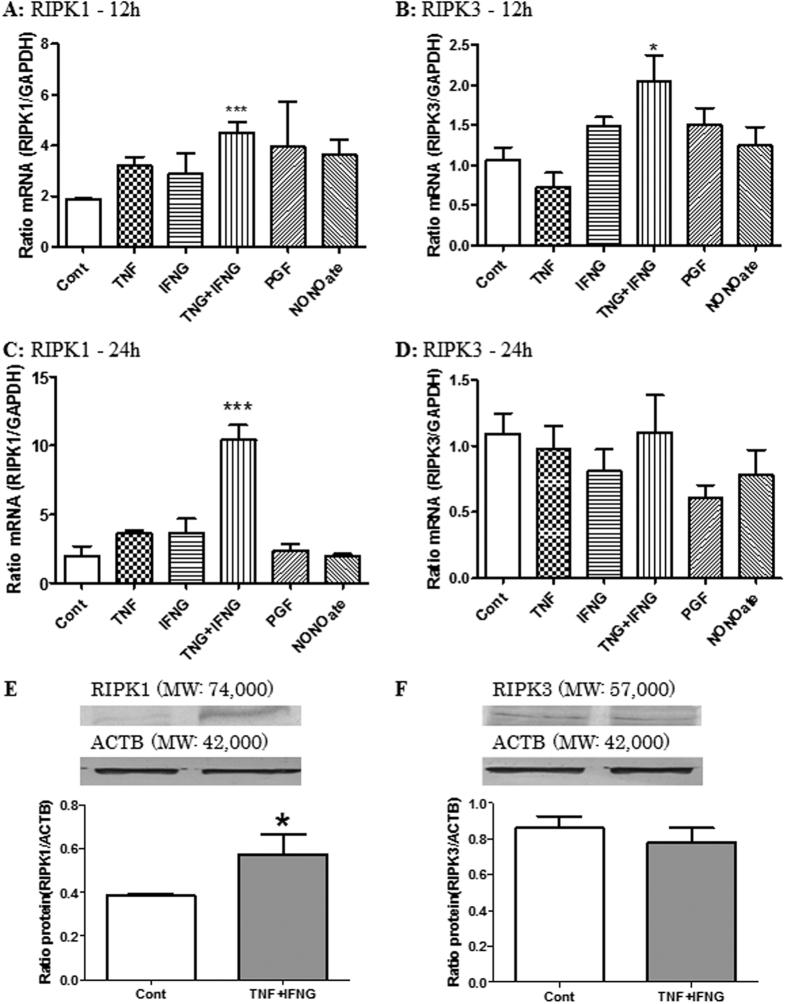
Confirmation of materials and adequate time to influence *RIPK1* and *RIPK3* mRNA and expression effects of TNF and IFNG on the expression of RIPK1 and RIPK3 protein in cultured luteal steroidogenic cells (LSCs). LSCs were treated with TNF (2.3 nM) and/or IFNG (2.5 nM), PGF (1.0 μM) or NONOate (100 μM) for 12 and 24 h. (**A**) and (**C**) show the expression of *RIPK1* mRNA, and (**B**) and (**D**) show the expression of RIPK3 in the LSCs after culture for 12 and 24 h, respectively. Data are expressed as the relative ratio of *RIPK1* or *RIPK3* mRNA to *GAPDH* mRNA levels. All values are the means ± SEM. All of the experiments were repeated more than three times. The data were statistically analyzed by ANOVA followed by Dunnett’s multiple comparison test. Asterisks indicate significant differences compared with control (*P < 0.05, ***P < 0.01, respectively). (**E**) and (**F**) show representative western blot bands for RIPK1 or RIPK3 and ACTB in cells treated with TNF (2.3 nM) and IFNG (2.5 nM) for 24 h. The resulting signal was visualized using the alkaline phosphatase visualization procedure and quantitated by computer-assisted densitometry. Data are expressed as the relative ratio of RIPK1 or RIPK3 protein to ACTB protein. All values are the means ± SEM. All of the experiments were repeated more than three times. The data were statistically analyzed by Student’s t-test. Asterisks indicate significant differences compared with control for 24 h (P < 0.05).

To investigate the regulator for the RIPKs expression in bovine LSCs, cells were treated with several luteolytic factors 12 or 24 h. Then real-time RT-PCR and western blotting were performed. Treatment with TNF in combination with IFNG highly up-regulated the expression of *RIPK1* mRNA (Fig. 5A and C; up to 243% and 528% of the control at 12 and 24 h, respectively; P < 0.05). *RIPK3* mRNA was up-regulated by TNF + IFNG at 12 h (Fig. 5B; up to 323% of control value; P < 0.05). However, this effect was no longer evident in the cells after 24 h incubation with the cytokines (Fig. 5D; P > 0.05). The NO donor and PGF did not affect directly *RIPKs* expression at the gene level at any time of treatment (P > 0.05). Therefore, in the next study at the protein level, only the combined treatment with TNF and IFNG was used.

Treatment with TNF in combination with IFNG up-regulated the expression of RIPK1 protein but not RIPK3 protein in LSCs cultured for 24 h (Fig. 5E and F; P < 0.05).

### Effects of Nec-1 on function of the cultured LSCs

**Figure 6:**
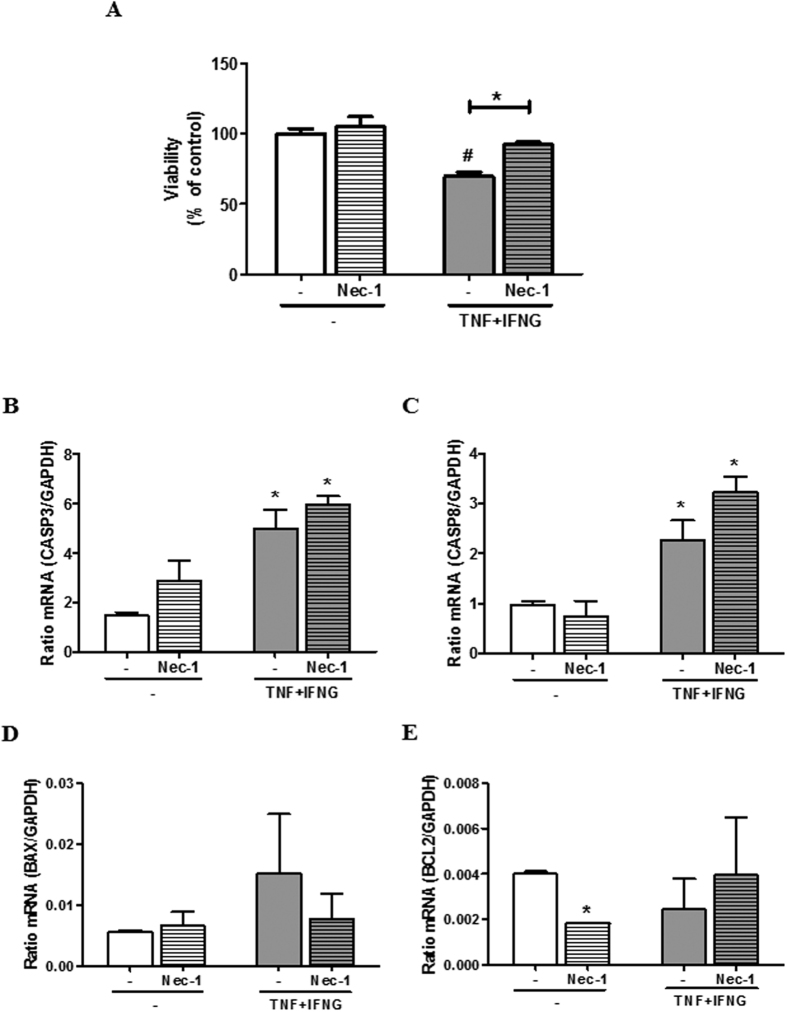
Effects of necrostatin-1 (Nec-1) on cell viability and expression of intracellular apoptosis related factors in luteal steroidogenic cells (LSCs). (**A**) The cells were treated with TNF (2.3 nM) + IFNG (2.5 nM) in combination with Nec-1 (50 μM) for 24 h. After culture, cell viability was measured. In this assay, data were expressed as percentages of the appropriate control values. All values are the means ± SEM. All experiments were repeated more than three times. The data were statistically analyzed by Student’s t-test. Asterisk (*) indicates significant differences (P < 0.05) compared to control, and hash (#) indicates significant differences between the presence and absence of Nec-1 (P < 0.05). Comparison of relative amounts of *CASP3* (**B**), *CASP8* (**C**), *BAX* (**D**) or *BCL2* (**E**) mRNA determined by quantitative RT-PCR in the LSC after treatment with Nec-1 (50 μM) and/or TNF (2.3 nM) + IFNG (2.5 nM) for 12 h. All experiments were repeated more than three times. The data were statistically analyzed by Student’s t-test. Asterisk (*) indicates significant differences compared to control (P < 0.05).

To clarify the role of RIPKs on function in LSCs, cells were exposed to Nec-1, which is an inhibitor for RIPK1 activity, with or without TNF + IFNG. Thereafter, cell viability and P4 secretion were measured. While a single treatment with Nec-1 did not affect LSCs viability, it prevented cell death induced by TNF + IFNG in the LSCs (Fig. 6A). Nec-1 did not affect P4 production (Supplementary Fig. 2).

**Figure 7:**
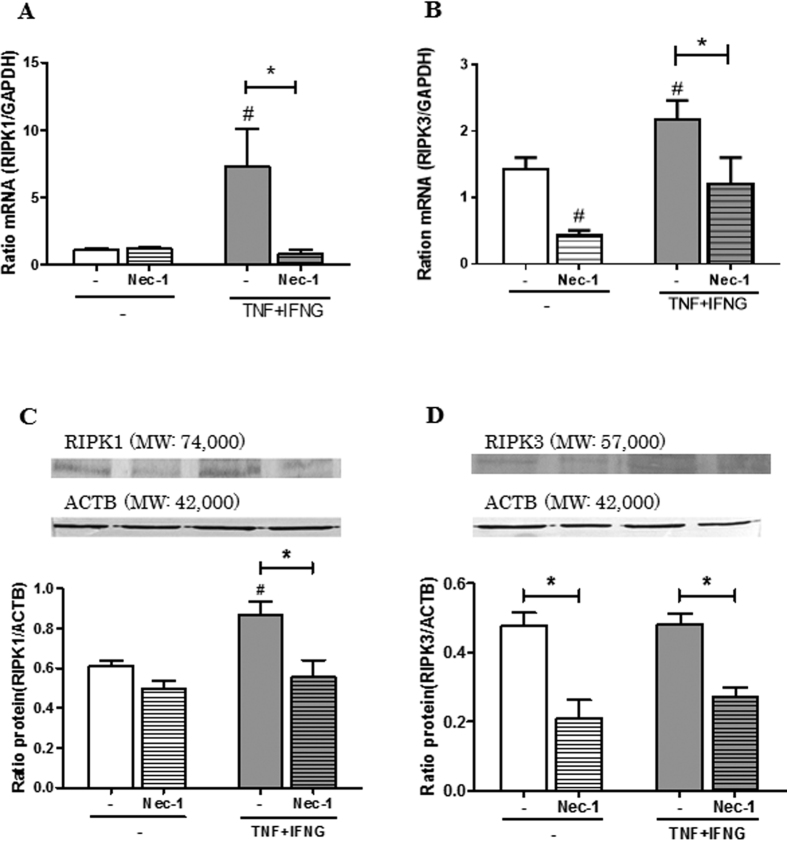
Effects of Nec-1 on the expression of RIPK1 and RIPK3 mRNA and protein in luteal steroidogenic cells (LSCs). (**A**) and (**B**) Comparison of relative amounts of RIPK1 or RIPK3 mRNA determined by quantitative RT-PCR in bovine LSCs after treatment with Nec-1 (50 μM) and/or TNF (2.3 nM) + IFNG (2.5 nM) for 12 h. (**C**) and (**D**) Representative western blot bands for RIPK1 or RIPK3 and ACTB in cells treated with Nec-1 (50 μM) and/or TNF (2.3 nM) + IFNG (2.5 nM) for 24 h. The resulting signal was visualized using the alkaline phosphatase visualization procedure and quantitated by computer-assisted densitometry. Data are expressed as the relative ratio of RIPK1 or RIPK3 protein to ACTB protein. All values are the means ± SEM. All experiments were repeated more than three times. Asterisk (*) indicates significant differences compared to control (P < 0.05), and hash (#) indicates significant differences between the presence and absence of Nec-1 (P < 0.05).

To confirm the pathway of cell death, i.e., apoptotic pathway or necroptotic pathway, real-time PCR and western blotting were performed after treatment of Nec-1 with or without TNF + IFNG in LSCs. Nec-1 did not affect TNF + IFNG-induced *CASP-3* and *CASP-8* expression (Fig. 6B and C). *BCL2* mRNA expression was down-regulated by a single treatment with Nec-1 (Fig. 6E). TNF and IFNG-induced RIPK1 mRNA and protein expression was down-regulated by Nec-1 (Fig. 7A and C; P < 0.05). Furthermore, TNF + IFNG-induced RIPK3 mRNA and protein expression was down-regulated by Nec-1 treatment (Fig. 7B and D; P < 0.05). Full length lanes of western blotting are shown in Supplementary Fig. 3.

## Discussion

Until now, there were no reports indicating that caspase-independent cell death can occur in the bovine CL during the course of luteolysis. In the present study, we proposed a new luteolytic mechanism responsible for steroidogenic cell death and elimination from bovine CL, i.e., RIPK-dependent necroptosis. Caspase-dependent apoptosis, which is induced by binding of death ligands to death receptors on the cell membrane, is known as a typical programmed cell death mechanism^16^,^17^ Cell-death receptors such as TNFR super family receptors are activated by binding death ligands. Thereafter, cytoplasm CASPs are recruited to death receptors and activated, and result in cell elimination during luteolysis *via* apoptosis^17^. In contrast, activated death receptors are known to recruit not only CASPs but also RIPKs^25^,^26^,^28^. After recruitment of RIPKs by death receptors, if deubiquitination of RIPKs by intracellular factors, such as cylindromatosis (CYLD), occurs, RIPKs form a death-inducing signaling complex (DISC) II that results in cell death by necroptosis^29^. In the present study, we have shown that RIPK1 and RIPK3 expression is strongly elevated in the bovine CL during spontaneous or PGF-induced luteolysis. Our results clearly showed that RIPKs-dependent necroptosis plays important roles in bovine structural luteolysis.

Cellular FLICE-like inhibitory protein (cFLIP) may be a candidate intracellular factor regulating RIPK activation and necroptosis^30^. Cellular FLIP was originally described as a regulator of death receptor-mediated apoptosis^31^. At the protein level, it occurs in two endogenous forms, cFLIP long (cFLIP_L_) and cFLIP short^32^, and it was reported that the CASP8-cFLIP_L_ complex inhibits not only apoptosis but also RIPK-dependent necroptosis^32^,^33^. Our recent report showed that cFLIP_L_ is expressed throughout the estrous cycle in bovine CL tissues and decreased in regressed CL^34^. These findings and the present study suggest that cFLIP_L_ down-regulates expression of RIPKs and inhibits necroptosis in the bovine CL during early to late luteal stages.

Detection of RIPKs localization in bovine LSCs and LECs by immunohistochemistry suggests the possibility that RIPKs-dependent necroptosis occurs in these cells. To clarify the mechanisms of necroptosis in these cells and during luteolysis, as the first step, we investigated the regulatory mechanisms of RIPKs expression in bovine LSCs. Since administration of PGF up-regulated both RIPK1 and RIPK3 *in vivo*, this may imply that PGF or some intra-luteal factors which mediate PGF action are crucial stimulators for RIPKs expression. The effect of PGF, which is known to be the most common luteolytic factor, was investigated both *in vivo* and *in vitro*, and the local effects of PGF on luteal function are very complex^2^,^3^,^13^,^28^,^35^,^36^. Based on the above findings, it is clear that the luteolytic effect of PGF is not directly upon LSCs and LECs to induce cell death, but it depends on cell composition and contact^37^. In the present study, PGF did not affect *RIPKs* mRNA expression in a pure population of cultured LSCs. Therefore, it could be concluded that the luteolytic action of PGF on bovine CL *in vivo* is mediated by several auto/paracrine factors which activate luteolytic mechanisms responsible for structural and functional luteal regression^38^. Similarly, the effect of PGF on RIPKs expression might depend on some mediators or upon cell composition and contact. Nitric oxide induces luteolysis *in vivo*, and stimulates CASP3 activity and apoptosis in cultured LSCs^8^,^39^,^40^. On the other hand, NO inhibits CASP3 activity and apoptosis in various types of cells^41^,^42^,^43^. Thus, NO plays both roles, inhibiting or stimulating programmed cell death depending on the cell type. In the present study, we investigated the effect of NO on RIPKs expression, and an NO donor, NONOate, did not affect mRNA expression of both *RIPK1* and *RIPK3*. The experimental design that we used in the present study could not clarify the effect of PGF and NO on RIPKs expression in LSCs. Further studies are needed to determine the effects of NO as well as PGF on RIPKs expression and necroptosis in bovine LSCs.

During luteolysis, it is known that the number of immune cells (e.g., T lymphocytes, macrophages) increases in the bovine CL^44^. The immune cells produce a variety of cytokines, including TNF and IFNG, which have been shown to induce functional and structural luteolysis in the cow^2^,^9^,^45^,^46^. It has been found that TNF superfamily ligands (FAS ligand and TNF) can stimulate RIPK-dependent necroptosis in several types of cells such as human T cells and mouse dendritic cells^24^,^25^,^26^. However, treatment with TNF alone did not stimulate *RIPKs* mRNA expression in the present study. Furthermore, although type I IFNs, such as IFN α and β, can stimulate necroptosis in cancer cells and macrophages^47^,^48^, a single treatment with IFNG also did not affect *RIPKs* mRNA expression in bovine LSCs. IFNG induces expression of TNF superfamily receptors in several cells^10^,^49^,^50^ including bovine LSCs^7^, and the combination of TNF and IFNG can stimulate acute apoptosis and luteolysis^7^,^10^,^51^. Based on the above findings, we hypothesized that TNF in combination with IFNG can stimulate RIPK-dependent cell death. As expected, treatment with TNF in combination with IFNG increased *RIPK1* mRNA expression significantly at 12 and 24 h. Moreover, TNF + IFNG upregulated RIPK1 protein expression, suggesting that TNF is a crucial regulator for RIPK1 as well as IFNG in bovine LSCs. Although *RIPK3* mRNA was up-regulated by TNF and IFNG at 12 h, RIPK3 protein did not increase after treatment with TNF and IFNG. This contradiction is thought to be caused by the time lag between transfer and translation or adjustment at the translation stage. Thus, the combination of TNF and IFNG was revealed as an important stimulator for RIPKs, particularly RIPK1, expression in bovine LSCs.

Moreover, for final confirmation that RIPKs-dependent cell death is involved in TNF + IFNG-induced death in LSCs, we used Nec-1 which inhibits the kinase activity of RIPK1 and prevents RIPK1/RIPK3 interaction^21^,^24^. Apoptosis occurs through two main signaling cascades, namely, the extrinsic apoptotic route and intrinsic apoptotic cascade^14^,^15^. Some studies have revealed that TNF and IFNG strongly stimulate the extrinsic apoptotic route *via* stimulating several cytokine receptors, such as TNFRI and FAS, in bovine LSCs^6^,^7^,^9^,^10^. Furthermore, another major apoptotic pathway, the intrinsic apoptotic cascade, is regulated by members of the Bcl-protein family, which is composed of antiapoptotic (*e.g*., Bcl-2) and proapoptotic (*e.g*., Bax) proteins^4^,^15^,^52^. In both the extrinsic and intrinsic apoptotic routes, CASP3 is a pivotal executioner for apoptosis^18^. In the present study, Nec-1 prevented spontaneous *Bcl-2* mRNA expression, but not expression of TNF + IFNG-induced *CASP8* and *CASP3* mRNA, revealing that Nec-1 did not affect CASPs-dependent apoptosis in bovine LSCs. Additionally, while Nec-1 did not affect *CASP3* mRNA expression, it could rescue LSCs from TNF + IFNG-induced cell death, strongly suggesting that the RIPKs-dependent necroptosis pathway is involved in TNF + IFNG-induced cell death in bovine LSCs. In addition, TNF is also reported to stimulate cell death in human luteal cells^53^ and luteal cell death induces the luteolysis in human^51^. It was demonstrated that TNF-induced cell death is apoptosis^53^ and it has never reported the presence of necroptosis in human luteal cells. The present results may contribute to the development of research for cell death of luteal cells and luteolysis in human.

Furthermore, Nec-1 increased cell viability but not P4 secretion, suggesting that Nec-1 prevention of cell death is not mediated/modulated by P4, which prevents apoptosis in bovine LSCs^3^,^11^,^54^. Taken together, TNF + IFNG-induced cell death in bovine LSCs may be regulated not only by CASP-dependent apoptosis but also by RIPK-dependent necroptosis. The results concerning expression of RIPK1 and RIPK3 invite us to examine details of the cascade of necroptosis in bovine LSCs. Generally, RIPK1 is thought to have a crucial role in recruiting and activating RIPK3^24^,^55^. Meanwhile, RIPK3 is also reported to be activated by TNF in the absence of RIPK1^28^, or that RIPK1 can inhibit RIPK3-dependent necroptosis^56^. The question whether RIPK1 is essential for regulation of RIPK3 expression in the bovine CL is still open. However, it has been shown in several reports that during RIPK-dependent necroptosis the activity of RIPKs is highly increased without any changes in their gene expression^20^,^22^,^23^,^24^,^55^,^56^. Thus, as a result of our findings it might be thought that, although RIPK1 does not affect RIPK3 expression in bovine LSCs, necroptosis is regulated by changes in RIPKs expression in these cells.

**Figure 8:**
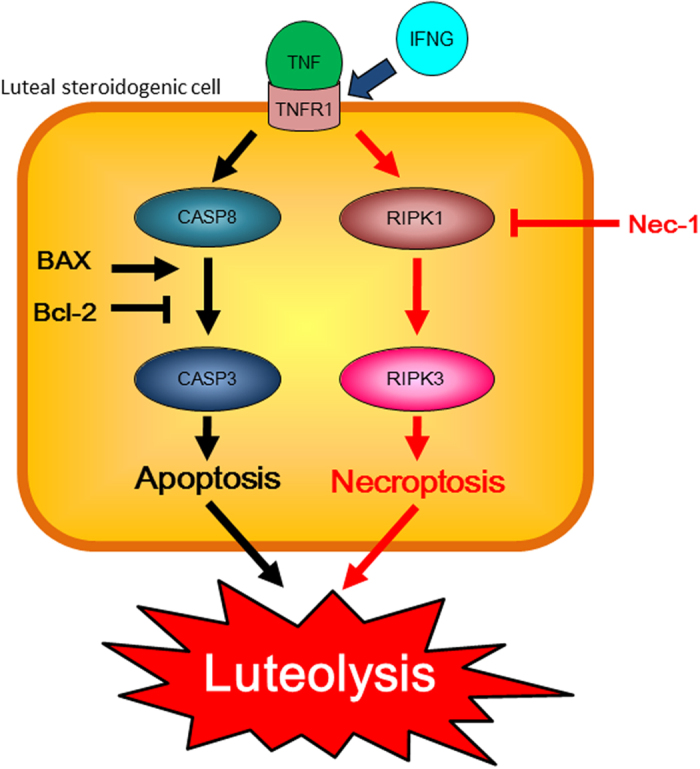
Schematic representation of possible mechanisms to induce cell death in the bovine LSCs. Pro-inflammatory cytokines, TNF and IFNG, can induce at least two cell death pathway, i.e., CASPs-dependent apoptotic pathway and RIPKs-dependent necroptotic pathway, in the bovine LSCs. Apoptotic pathway is mediated by CASP8 and CASP3, BAX and Bcl-2 can play as inducer and inhibitor of apoptotic pathway, respectively. In necroptotic pathway, RIPK1 and RIPK3 play central role to induce cell death. RIPK1 inhibitor, Nec-1, can inhibit necroptotic pathway. Both CASPs-dependent apoptosis and RIPKs-dependent necroptosis can be potent mechanisms of luteolysis in cattle.

This study demonstrated for the first time the expression of RIPK1 and RIPK3, and the presence of RIPKs-dependent cell death, in the bovine CL both *in vivo* and *in vitro*. These findings suggest that RIPKs-dependent cell death can be a potent mechanism of TNF- and IFNG-mediated CL regression in cattle. Schematic representation of possible mechanisms to induce cell death in the bovine LSCs is shown in Fig. 8.

## Methods

### Animal procedures

All *in vivo* experiments were carried out in accordance with the relevant guidelines: EU Directive of the European Parliament and the Council on the protection of animals used for scientific purposes (22 September 2010; No 2010/63/EU), Polish Parliament Act on Animal Protection (21 August 1997, Dz.U. 1997 nr 111 poz. 724) with further novelization - Polish Parliament Act on the protection of animals used for scientific or educational purposes (15 January 2015, Dz.U. 2015 poz. 266). All animal procedures were approved and accepted accordingly to relevant guidelines by the Local Ethics Committee for Experiments on Animals in Olsztyn, Poland (Agreement No. 85/2012).

For determination of expression of RIPKs throughout the estrous cycle, CLs from normally cycling cows were obtained from a local abattoir. Luteal stages were confirmed as being early (Days 2–3 after ovulation: n = 4), developing (Days 5–7: n = 4), mid (Days 10–12: n = 4), late (Days 15–17: n = 4) and regressed (Days 19–21: n = 4) by additional macroscopic observation of the ovary and uterus as described previously^57^.

For cell culture, ovaries with CLs (Day 10–12 of the cycle) were submerged in ice-cold physiological saline and transported to the laboratory.

To determine the effect of PGF on expression of RIPKs, ovaries with CL were taken out via vagina (colpotomy) using a Hauptner’s effeninator (Hauptner & Herberholz GmbH & Co. KG, Solingen, Germany) on Day 10 post-ovulation, i.e., non-treated (0 h, control: n = 4), and at 2- (n = 4), 4- (n = 4) and 12-h (n = 4) after injection of a luteolytic dose of PGF analog (dinoprost, i.m.: 5 mg; as recommended by the manufacturer) as described previously^58^, and corpora lutea were collected from these ovaries.

The CL tissue samples were then immediately placed into a 1.5 ml microcentrifuge tube containing either 0.4 ml TRI Reagent^®^ (Sigma–Aldrich Corp., St. Louis, MO, USA, #T9424) or nothing, homogenized, and stored at −80 °C until processed appropriately for mRNA and protein analysis.

For immunohistochemistry, pieces of CL tissues were fixed in 4% (vol/vol) neutral formalin (pH 7.4) for 20–24 h and then embedded in paraffin.

### Luteal steroidogenic cell isolation

The CLs at mid luteal stage were used for cell culture. Luteal tissue was enzymatically dissociated, and luteal cells were cultured as described previously^59^. Cell viability was greater than 85% as assessed by trypan blue exclusion. The cells in the suspension consisted of about 70% small LSCs, 20% large LSCs, 10% endothelial cells or fibrocytes, and no erythrocytes. In this study, these cells were defined as LSCs.

### Changes in RIPK1 and RIPK3 mRNA and protein expression levels throughout the estrous cycle and PGF-induced luteolysis *in vivo*

The expression of RIPK1 and RIPK3 mRNA and protein in the CL tissues of each stage (Early, Developing, Mid, Late and Regressed; each stage n = 4) and after PGF administration (0 h: control, 2 h, 4 h, and 12 h; each stage n = 4) were examined by quantitative RT-PCR and western blotting.

RIPKs protein localization in CL tissues throughout the estrous cycle (Early, Mid and Regressed; each stage n = 3) and after PGF administration (0 h: control, 4 h and 12 h; each stage n = 3) were analyzed by immunohistochemistry.

### Local regulatory mechanisms of RIPK1 and RIPK3 expression in cultured LSCs

To clarify regulatory factors and the timing of changes of RIPK1 and RIPK3 expression in cultured mid LSCs, the cells were exposed to 2.3 nM recombinant human TNF (Dainippon Sumitomo Pharma Co., Ltd., Osaka, Japan) and/or 2.5 nM recombinant bovine IFNG (kindly donated by Dr. S. Inumaru, NIAH, Ibaraki, Japan), 1.0 μM PGF (Sigma-Aldrich, #P7652) or 100 μM NONOate (a NO donor; Cayman Chemical, Ann Arbor, MI, USA, #82150) for 12 and 24 h. The doses for treatments were selected based on previous reports^7^,^8^,^11^,^13^. After culture, the expression of RIPKs mRNA was determined by quantitative RT-PCR.

In addition, RIPKs protein expression was assessed by western blotting in cells treated with TNF and IFNG for 24 h, based on data from the first part of the experiment.

### Effects of RIPKs on the function of cultured LSCs

To reveal the effects of Nec-1 on P4 production and cell death in LSCs, the cells were exposed to Nec-1 (50 μM; Enzo Life Sciences, Inc., NY, USA, #BML-AP339-0100) with or without TNF and IFNG treatments for 12 or 24 h. The Nec-1 dose was selected based on previous reports^60^ and a preliminary study (data not shown). After 12 h of stimulation, the concentration of P4 in culture media and mRNA expression of *BCL-2, BAX, CASP8, CASP3, RIPK1* and *RIPK3* were determined by EIA and quantitative RT-PCR, respectively. Furthermore, after 24 h of stimulation, cell viability and RIPKs protein expression were determined by AlamarBlue assay and western blotting, respectively.

### Real time PCR

Real time PCR was performed with an ABI 7900 HT sequence detection system using SYBR Green PCR master mix (Applied Biosystems, Foster City, CA, USA). The primer length (20–25 bp) and GC contents of each primer (50–60%) were synthesized (Supplementary Table 1). After a preliminary study, *GAPDH* was chosen as the best housekeeping gene. All primers were synthesized by Sigma (Custom Oligos, Sigma). Real time PCR was carried out as follows: initial denaturation (10 min at 95 °C), followed by 40 cycles of denaturation (15 s at 95 °C) and annealing (1 min at 60 °C). Data were analyzed using the method described by Zhao and Fernald^61^.

### Western Blotting

Briefly, CL tissues or cultured LSCs were collected on ice in RIPA buffer (Sigma-Aldrich, #R0258) in the presence of a protease inhibitor cocktail (Roche, Bazel, CHE, #11697498001). Each lysate was heated at 95 °C for 5 min and resolved using 10% SDS-PAGE, followed by transferred onto Immobilon-P Transfer Membrane (Millipore, MA, USA, #IPVH00010) in transfer buffer (0.3 mM Tris buffer, pH 10.4, 10% methanol; 25 mM Tris buffer, pH 10.4, 10% methanol; 25 mM Tris buffer, pH 9.4, 10% methanol, 40 mM glycine). After blocking in 5% non-fat dry milk in TBS-T buffer (Tris-buffered saline, containing 0.1% Tween-20) for 1.5 h at room temperature, the membranes were incubated overnight with rabbit polyclonal anti-RIPK1 or RIPK3 (dilution for both antibodies 1:1000; Sigma-Aldrich, #SAB3500420; Sigma-Aldrich, #SAB2102009); or mouse monoclonal anti-β-actin (ACTB) antibody (dilution for antibody 1:2000; Sigma-Aldrich, #A2228) at 4 °C. Subsequently, the membrane were incubated with secondary polyclonal anti-rabbit alkaline phosphatase-conjugated antibody (dilution 1:4000; for RIPK1 and RIPK3; Sigma-Aldrich, #A3812) or secondary anti-mouse IgG alkaline phosphatase-conjugated antibody (dilution 1:30,000) for ACTB (Sigma-Aldrich, #A3562) for 1.5 h at room temperature. After washing, immune complexes were visualized using the alkaline phosphatase visualization procedure. The intensity of the immunological reaction in the samples was estimated by measuring the optical density in the defined area by computerized densitometry using NIH Image (National Institutes of Health, Bethesda, MD, USA). Full length lanes of western blotting are shown in Supplementary Fig. 3.

### Immunohistochemistry

After dewaxing and washing, paraffin-embedded sections, cut at 4-μm thickness, were incubated at room temperature with 0.3% hydrogen peroxide in methanol for 20 min to inactive endogenous peroxidase. Then, the sections were washed in PBS and incubated with normal goat serum for 60 min at room temperature followed by RIPK1 (1:400) or RIPK3 (1:200) antibodies at 4 °C overnight. After washing twice, the sections were incubated with biotinylated anti-rabbit IgG (1:500; Vector Laboratories, CA, USA, #PK-6200) for 60 min at room temperature. The reaction sites were visualized using a Vectastain ABC Elite kit (Vector Laboratories, #PK-6200) for 60 min at room temperature and an ImmPACT 3,30-Diaminobenzidine (DAB) Peroxidase Substrate Kit (Vector Laboratories, #SK-4100) for 5 min. The sections were counterstained for 2 min with hematoxylin. Positive immunohistochemistry staining was assessed as a characteristic brown staining, using a light microscope (Olympus BX51, Tokyo, Japan).

### Cell viability

Viability of cells was determined by the AlamarBlue assay. Briefly, after culture, the culture medium was replaced with 100 μl D/F medium without phenol red, and 10 μl AlamarBlue^®^ solution (Thermo Fisher Scientific, MA, USA, #DAL1100) was added. The cells were incubated for 2 h at 37 °C. The optical densities of the supernatants were read at 540 and 620 nm on a microplate reader, while fluorescence was measured in arbitrary fluorescent units following excitation at 530–560 nm and emission at 590 nm. In this assay, data were expressed as percentage of the appropriate control values.

### Statistical Analysis

All statistical analyses and Graphic presentation of the data were done using GraphPad Software version 6, San Diego, USA. The results were considered significantly different when P < 0.05. The data are shown as the mean ± SEM of values obtained in separate experiments, each performed in quadruplicate.

In Experiment 1 (Figs 1 and 3), statistical analyses were performed using a non-parametric Kruskal-Wallis test followed by Dunnett’s multiple comparison test.

In preliminary *in vitro* studies (Experiments 2: Fig. 5A–D), statistical differences in RIPKs expression in LSCs on the gene level were examined using parametric one-way ANOVA followed by Dunnett’s multiple comparison test (comparing the treatment group with the controls). Statistical differences in expression of RIPKs on the protein level in LSCs between controls and the TNF + IFNG treatment group (Experiment 2, Fig. 5E and F) were analyzed using Student’s t-test.

In Experiment 3 (Figs 6 and 7), differences in effects of Nec-1 on LSCs in the control and TNF + IFNG treatment groups were analyzed using a two-way ANOVA test followed by the Bonferroni comparison test.

## Additional Information

**How to cite this article**: Hojo, T. *et al*. Programmed necrosis - a new mechanism of steroidogenic luteal cell death and elimination during luteolysis in cows. *Sci. Rep.* **6**, 38211; doi: 10.1038/srep38211 (2016).

**Publisher’s note:** Springer Nature remains neutral with regard to jurisdictional claims in published maps and institutional affiliations.

## Supplementary Material

Supplementary Information
