# Supplementary material for: Programmed necrosis - a new mechanism of steroidogenic luteal cell death and elimination during luteolysis in cows
Source: Sci Rep. 2016 Nov 30;6:38211. doi: 10.1038/srep38211 (PMC5128806; doi:10.1038/srep38211)
Supplement: Supplementary Information [file srep38211-s1.pdf]

Supplementary information

Programmed necrosis - a new mechanism of steroidogenic luteal cell death and elimination during luteolysis in cows

Takuo Hojo, Marta J. Siemieniuch, Karolina Lukasik, Katarzyna K. Piotrowska-Tomala, Agnieszka W. Jonczyk, Kiyoshi Okuda, Dariusz J. Skarzynski

Supplementary Materials and Methods: (Page 2)

Supplementary Figure 1: Expression of RIPK1 and RIPK3 mRNA in the cultured LSC. (Page 3)

Supplementary Figure 2: Effects of necrostatin-1 (Nec-1) on P4 production in luteal steroidogenic cells (LSCs). (Page 4)

Supplementary Figure 3: Full length lanes of western blotting. (Page 5)

Supplementary Table 1: Sequences for primers and accession numbers for genes. (Page 6)

Supplementary references: (Page 7)

Reverse transcription PCR

Reverse transcription (RT)-PCRs were carried out with β-actin (ACTB), GAPDH, 3β-HSD, eNOS, RIPK1 and RIPK3 primers. The sequence of each primer is shown in supplementary Table 1 and they were used as housekeeping genes (GAPDH and ACTB), markers for LSCs (3β-HSD), or marker for LECs (eNOS). As a negative control (N.C.), PCR was performed without any primers. Each PCR yielded only a single amplification product. The PCRs were carried out using an RED Taq ReadyMix PRC Reaction Mix (Sigma-Aldrich, #R2523) and a thermal cycler (BioRad, Hercules, CA, USA). The conditions for the PCRs were as follows: after activation of DNA polymerase by incubating for 7 min at 94 C, 30 cycles of reactions including denaturation for 1 min at 94 C, annealing for 2 min at 55 C, and finally extension for 3 min at 72 C were performed. The PCR amplification was calibrated to determine the optimal number of cycles that would allow detection of the appropriate mRNA transcripts while still keeping amplification of these genes in the log phase. A two-fifths aliquot of each reaction mixture was electrophoresed on a 1.5% agarose gel containing ethidium bromide and photographed under ultraviolet illumination.

Hormone Determinations

Concentrations of P4 were determined directly from the cell culture media by direct enzyme immunoassay. As described previously[1] for P4 concentration assessment, antiserum was used at a final dilution of 1:100,000. HRP-labeled P4 was used at a final concentration of 1:75,000. The standard curve ranged from 0.39 to 100 ng/ml, and the concentration of P4 at 50% binding (ED50) was 4.3 ng/ml. The intra- and inter-assay coefficients of variation (CVs) were 5.6% and 8.8%, respectively.

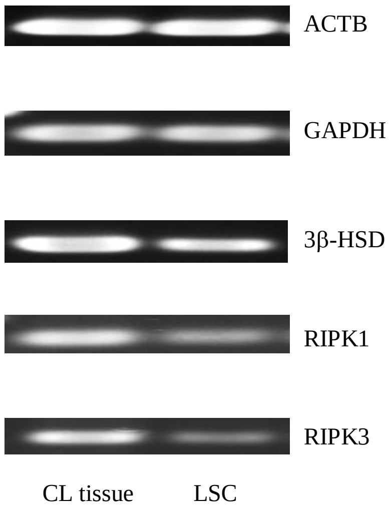

Supplementary Figure 1

Expression of *RIPK1* and *RIPK3* mRNA in bovine CL tissues and cultured LSCs

Representative RT-PCR bands of *RIPK1* and *RIPK3* mRNA in bovine CL tissues (lane 1) and isolated luteal steroidogenic cells (LSCs: lane 2).

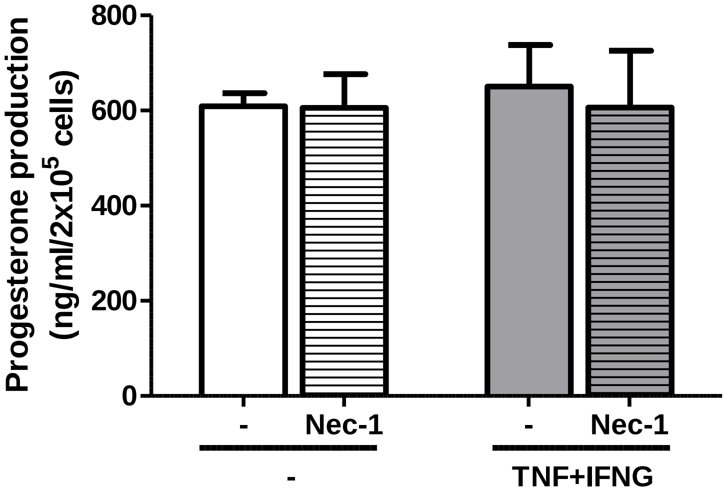

Supplementary Figure 2

Effects of necrostatin-1 (Nec-1) on P4 production in LSCs

The cells were treated with TNF (2.3 nM) + IFNG (2.5 nM) in combination with Nec-1 (50 μM) for 6 h. After culture, P4 concentrations in the supernatants were measured.

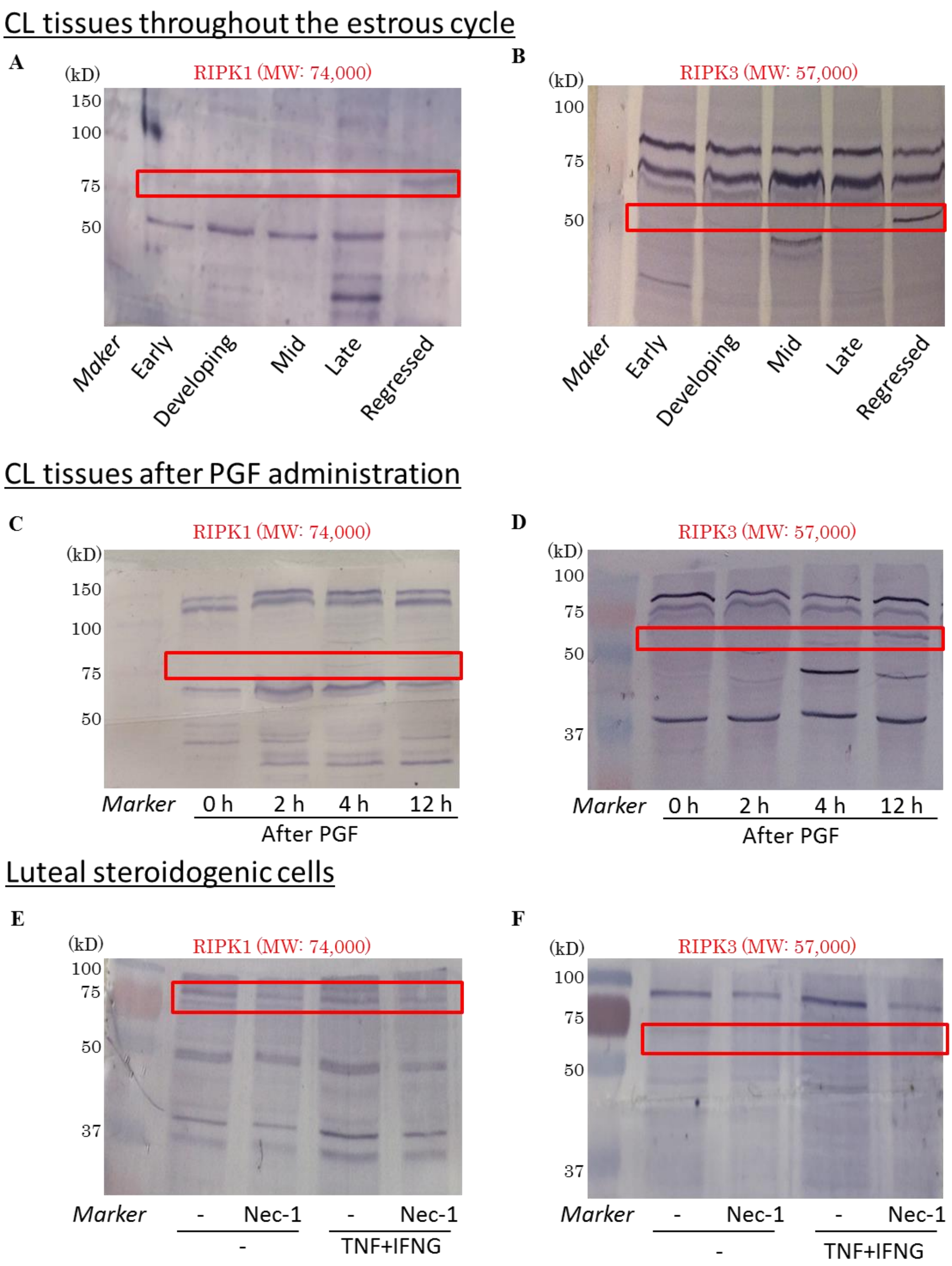

Supplementary Figure 3

Full length lanes of western blotting

Bands surrounded by red square were quantitated in figure 1 (A and B), figure 2 (C and D) and figure 7 (E and F), respectively.

gene	primer sequence	GenBank	PCR products size
RIPK1	5' GCAATAGCTCCAAGCAGGTC '3<br>5' TGTGCAGCAGGAAGTCATTC '3	NM_001035012	148 bp
RIPK3	5' CCAGAGAGAGCAGGTTCCAC '3<br>5' AATCAGGCGGTTGTTGTTTC '3	NM_001101884.2	219 bp
3βHSD	5' CTAATGGGTGGGCTCTGAAA '3<br>5' CACGCTGTTGGAAAGAGTCA '3	NM_174343	473 bp
CASP3	5' TGGTGCTGAGGATGACATGG '3<br>5' GAGCCTGTGAGCGTGCTTTT '3	NM_001077840.1	163 bp
CASP8	5' CTGAGAGAAGAGGCCCGTGA '3<br>5' CCCGGCTTAGGAACTTGAGG '3	DQ319070.1	173 bp
BAX	5' GTGCCCGAGTTGATCAGGAC '3<br>5' CCATGTGGGTGTCCCAAAGT '3	U92569.1	126 bp
BCL2	5' GAGTTCGGAGGGGTCATGTG '3<br>5' GCCTTCAGAGACAGCCAGGA '3	U92434.1	203 bp
GAPDH	5' CACCCTCAAGATTGTCAGCA '3<br>5' GGTCATAAGTCCCTCCACGA '3	BC102589	103 bp
ACTB	5' GAGGATCTTCATGAGGTAGTCTGTCAGG '3<br>5' CAACTGGGACGACATGGAGAAGATCTGGCA '3	AY141970	349 bp

Supplementary Table 1 Sequences for primers and accession numbers for genes

Supplementary References

1. Korzekwa, A. *et al.* Effects of prostaglandin F2α and nitric oxide on the secretory function of bovine luteal cells. *J Reprod Dev.* **50**, 411-417 (2004)
